# Successful Treatment of BRAF V600E-Mutant Intrahepatic Cholangiocarcinoma With Encorafenib, Binimetinib, and Cetuximab

**DOI:** 10.7759/cureus.86263

**Published:** 2025-06-18

**Authors:** Toshifumi Yamaguchi, Yoshinobu Hirose, Yasutaka Nakazawa, Akiko Kagotani, Hiroyuki Kodama, Hiroki Yukami, Shin Kameishi, Nanako Matsuo, Elham Fakhrejahani, Hiroki Nishikawa

**Affiliations:** 1 Cancer Chemotherapy Center, Osaka Medical and Pharmaceutical University Hospital, Osaka, JPN; 2 Department of Pathology, Osaka Medical and Pharmaceutical University Hospital, Osaka, JPN; 3 Department of Oncology, Kyoto Breast Cancer Research Network, Kyoto, JPN; 4 Department of Second Internal Medicine, Osaka Medical and Pharmaceutical University Hospital, Osaka, JPN

**Keywords:** billiary tract cancer, binimetinib, braf v600 mutation, encorafenib, next generation sequencing (ngs)

## Abstract

BRAF mutations, particularly BRAF V600E, are rare but clinically significant molecular alterations in intrahepatic cholangiocarcinoma (IHCC), often linked to aggressive disease and poor prognosis. While targeted therapies have shown efficacy in BRAF-mutant colorectal cancer, their role in IHCC remains uncertain. This report describes the case of a 59-year-old woman with advanced BRAF V600E-mutated IHCC (cT3N3M1c, stage IVc) treated with encorafenib, binimetinib, and cetuximab. The BRAF V600E mutation was identified through next-generation sequencing. Initially, the patient was given gemcitabine, cisplatin, and durvalumab as first-line chemotherapy; however, her metastatic lesions progressed rapidly within one month. Due to the lack of response, second-line treatment with encorafenib, binimetinib, and cetuximab was started, resulting in a favorable initial response. Unfortunately, the disease progressed five months after initiating this regimen. This case highlights the potential benefit of combined BRAF/MEK/EGFR inhibition in BRAF V600E-mutated IHCC and underscores the need for further research to optimize treatment strategies for this patient population.

## Introduction

Biliary tract cancer (BTC) comprises a heterogeneous group of aggressive malignancies characterized by poor prognosis and limited treatment options [1,2]. While BRAF mutations have been established as important prognostic biomarkers in melanoma, they have also emerged as predictive biomarkers due to the availability of targeted therapies, indicating the potential efficacy of these treatments [3,4]. Other cancers, including BTC, may similarly benefit from therapies targeting BRAF mutations.

Although systemic chemotherapy remains the standard first-line treatment for BTC, recent advances in molecular profiling have identified potentially actionable mutations in some patients, opening new avenues for targeted therapy. Among these, BRAF mutations, particularly BRAF V600E, are found in approximately 5-7% of BTC cases and are associated with aggressive disease and poor outcomes [5,6].

A clinical trial demonstrated promising antitumor activity of dabrafenib (a BRAF inhibitor) and trametinib (a MEK inhibitor) in patients with various solid tumors harboring BRAF V600E mutations [7]. In BRAF V600E-mutated colorectal cancer, the combination of encorafenib, binimetinib, and the EGFR inhibitor cetuximab has shown significant clinical activity, leading to FDA approval of this treatment regimen.

Mechanistically, encorafenib selectively inhibits the mutant BRAF V600E kinase, thereby suppressing aberrant MAPK signaling. Binimetinib enhances this effect by inhibiting downstream MEK, further blocking the MAPK pathway. Cetuximab, an anti-EGFR monoclonal antibody, counteracts compensatory EGFR-mediated reactivation of the pathway, a known resistance mechanism when using BRAF inhibitors alone in epithelial tumors. This vertical inhibition of the MAPK pathway at multiple levels, BRAF, MEK, and EGFR, provides more sustained suppression of tumor-promoting signals, resulting in improved antitumor efficacy.

The triplet regimen of encorafenib, binimetinib, and cetuximab has shown a higher response rate than the doublet regimen of encorafenib and cetuximab; however, overall survival (OS) outcomes were similar. As a result, only the doublet regimen has received FDA approval, while the triplet combination has been approved in Japan for use in patients with various BRAF-mutated colorectal cancers [8-10].

Despite its potential, experience with this triplet regimen in BRAF-mutant BTC is still limited, with only a few cases reported in the literature [11-13]. This report presents a case of advanced BRAF-mutant BTC that showed a remarkable response to the combination of encorafenib, binimetinib, and cetuximab, highlighting the potential benefit of this targeted approach in molecularly selected BTC. This case contributes to the growing body of evidence supporting molecular profiling and targeted therapy in BTC and suggests a potential role for BRAF/MEK/EGFR inhibition in this rare molecular subset.

This article was previously posted to the Research Square preprint server on May 15, 2025.

## Case presentation

A 59-year-old woman with a history of cataracts and immune thrombocytopenic purpura had been in good health until January 2022, when she began experiencing occasional localized abdominal pain. She consulted her general practitioner, who recommended abdominal and pelvic CT to rule out diverticulitis. The CT, performed in February 2022, incidentally revealed a pancreatic mass and several hypodense lesions in both lobes of the liver, the largest in the right lobe measuring approximately 10 × 8.4 cm. The patient was referred to our hospital for further evaluation. She subsequently underwent upper esophagogastroduodenoscopy. Colonoscopy revealed suspected infiltration in the ileocecal region due to peritoneal metastasis. Biopsies were taken from the area of colonic invasion. Histopathology revealed adenocarcinoma at the site of invasion with peritoneal dissemination. Contrast-enhanced CT of the chest and pelvis showed a pancreatic tumor, peritoneal dissemination, lung metastases, and a liver mass.

**Figure 1:**
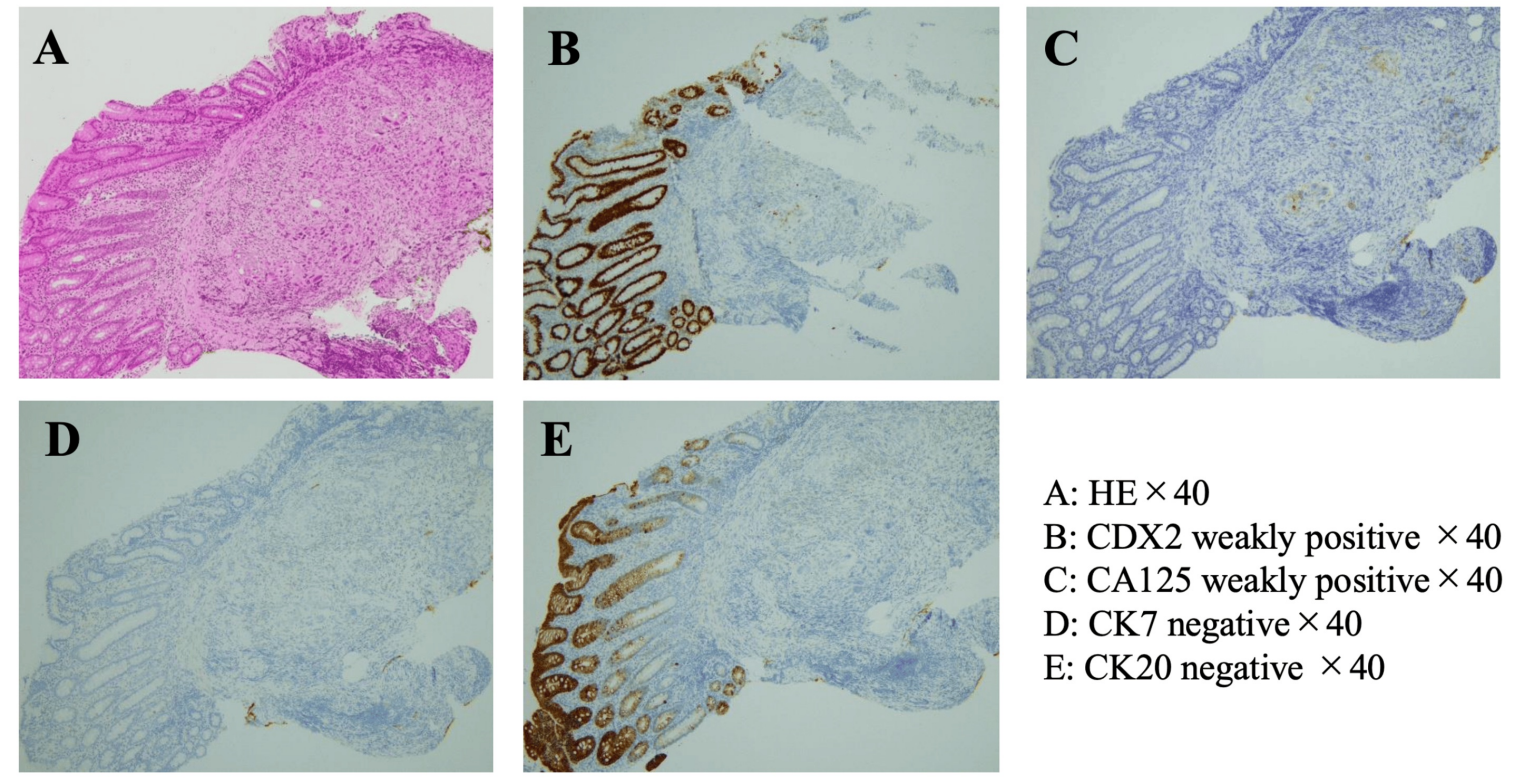
Histopathological assessment of the tumor (A) Dense infiltration of atypical epithelial cells was observed predominantly in the submucosa, with no involvement of the mucosal layer — a histological pattern consistent with metastatic carcinoma (hematoxylin and eosin staining; magnification ×40). (B-E) Immunohistochemical findings did not support a diagnosis of primary colorectal cancer: CDX2 was weakly positive, and CK20 was negative. CK7 was also negative, which is atypical for IHCC; however, CA125 was weakly positive, a marker often expressed in biliary tract tumors. Taking into account the patient’s clinical history and imaging findings, the lesion was diagnosed as a metastasis from the known bile duct carcinoma.

**Figure 2:**
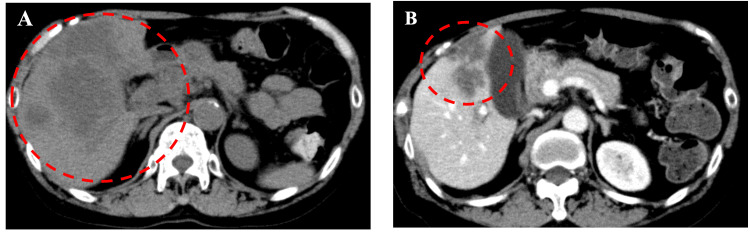
CT images demonstrating the initial response to combination therapy with encorafenib, binimetinib, and cetuximab (A) CT scan showing progression of the primary tumor after two cycles of treatment with durvalumab, gemcitabine, and cisplatin. (B) CT scan showing marked tumor shrinkage following two cycles of treatment with encorafenib, binimetinib, and cetuximab.

¹⁸F-fluorodeoxyglucose (FDG) PET/CT demonstrated multiple FDG-avid hypermetabolic lesions in the liver (SUVmax, 9.8), the largest measuring 10 cm in diameter. FDG accumulation was also observed in numerous enlarged abdominal lymph nodes and multiple nodules in both lungs. Endoscopic ultrasonography favored intrahepatic cholangiocarcinoma (IHCC) over pancreatic cancer, and bile duct biopsy confirmed adenocarcinoma (Figure 1). In the colon biopsy specimens, the surface epithelium appeared intact and showed no atypia. However, a dense infiltration of atypical epithelial cells was predominantly found in the submucosa, with no involvement of the mucosal layer. The tumor tested positive for CK7 and negative for CK20 and CDX2, supporting the diagnosis of metastatic IHCC rather than primary colorectal cancer. The histological findings were consistent with metastatic carcinoma. Taking into account the patient’s clinical history and imaging results, the colonic lesion was diagnosed as a metastasis from the known bile duct carcinoma. Consequently, the patient was diagnosed with IHCC with liver and lung metastases and peritoneal dissemination (Union for International Cancer Control 8th edition; cT3N3M1c, stage IVc; Figure 2).

After diagnosis, the patient was started on durvalumab (1500 mg) on day 1, along with gemcitabine (1000 mg/m²) and cisplatin (25 mg/m²) on days 1 and 8, as first-line chemotherapy. Next-generation sequencing (NGS) was performed using the FoundationOne CDx panel on a biopsy specimen from the peritoneal dissemination obtained at diagnosis. The specimen used for NGS was taken from the tumor lesion infiltrating the colon.

After two cycles of durvalumab, gemcitabine, and cisplatin, the patient developed abdominal pain. Treatment was discontinued, and a CT scan revealed rapid progression of liver metastases and the appearance of ascites. NGS identified mutations in BRAF V600E, FGF14 T229M, and TP53 splice site 376-1G>A. At the time, BRAF and MEK inhibitors had not yet been approved for BTC in Japan, although they had been recognized globally for use in BRAF V600E-mutated solid tumors. In this case, treatment was administered based on the recommendation of the institutional expert panel following comprehensive genomic profiling. After a multidisciplinary review by our oncology team, it was determined that the malignancy might be susceptible to BRAF and MEK inhibition, as well as their combination with anti-EGFR antibody therapy, following the same rationale as BRAF mutation-positive colorectal cancer.

Given the ineffectiveness of the durvalumab, gemcitabine, and cisplatin regimen, the patient was initiated on second-line therapy with encorafenib (300 mg/day), binimetinib (90 mg/day), and cetuximab (400 mg/m² as the initial dose, followed by 250 mg/m² weekly). A repeat CT scan in March 2022 (within six weeks of starting treatment) showed spontaneous resolution of ascites and significant shrinkage of multiple lung and lymph node metastases. Liver metastases and shrinkage at the primary site were particularly notable: the large right lobe lesion reduced from 6.0 × 4.6 cm to 3.2 × 2.7 cm, and the medium right lobe lesion from 5.2 × 2.4 cm to 2.6 × 2.0 cm. The patient’s abdominal pain improved rapidly and eventually resolved.

**Figure 3:**
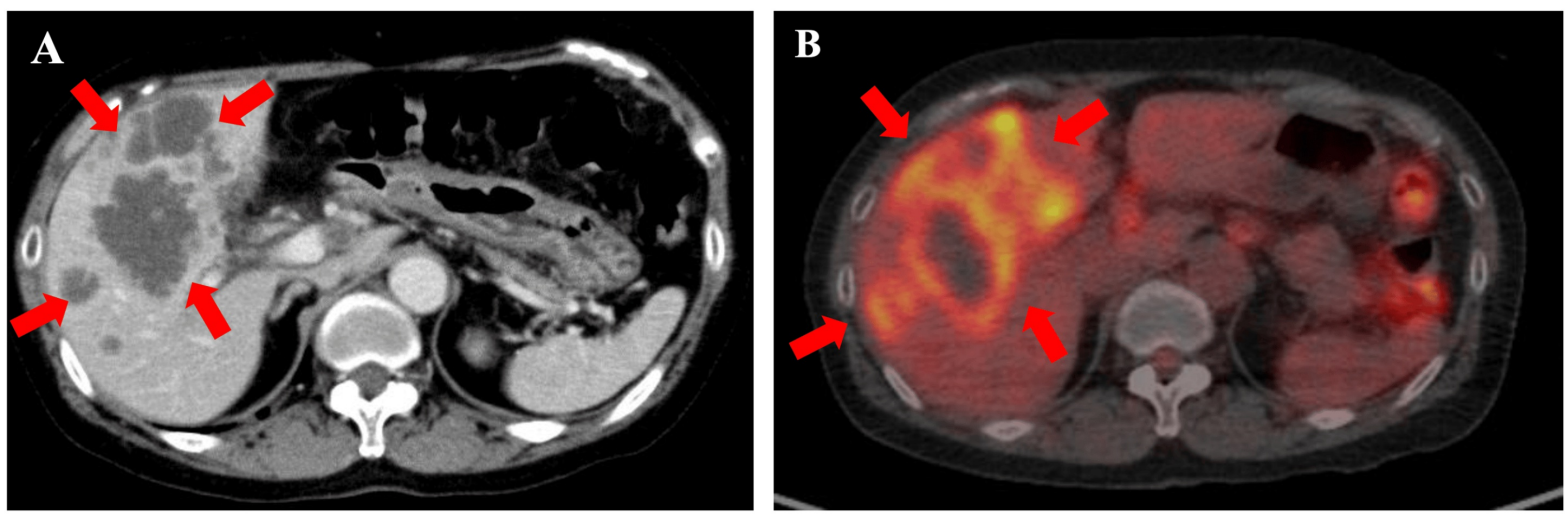
Baseline CT and PET-CT images of the abdomen before treatment (A) CT scan showing a large, predominantly hypodense mass measuring 10 × 8.4 cm in the right hepatic lobe at presentation. (B) Baseline PET-CT scan demonstrating a nodule in the right hepatic lobe with increased uptake of ¹⁸F-FDG. FDG, fluorodeoxyglucose

Two months later, in May 2022, radiological assessment via chest and pelvic CT showed a partial response (>50% shrinkage; Figure 3). As the metastatic lesions in the liver, peritoneum, and lungs were well controlled, treatment with encorafenib, binimetinib, and cetuximab was continued. The only observed adverse event was Grade 1 fatigue, based on the Common Terminology Criteria for Adverse Events (version 5.0). No other serious adverse events occurred.

**Figure 4:**
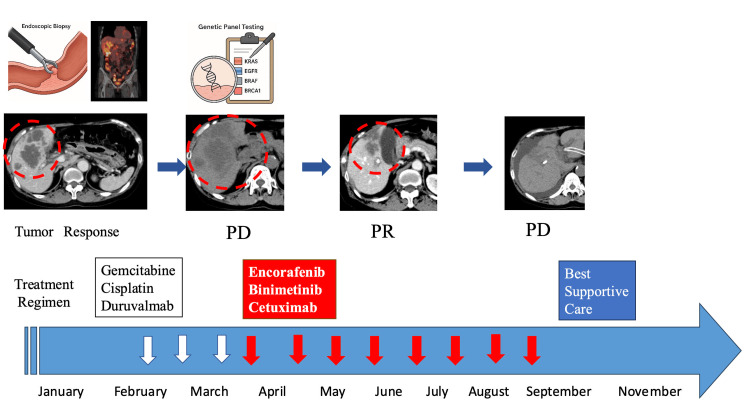
Treatment timeline of the patient described in this case

However, a follow-up CT scan performed four months later, in September 2022, revealed a rapid increase in the size of the liver metastases. Given the lack of efficacy of ongoing chemotherapy and the patient’s overall condition, the treatment strategy was shifted to supportive care. The patient died at home two months later, in November 2022 (Figure 4).

## Discussion

This case demonstrates the potential efficacy of targeted therapy with encorafenib, binimetinib, and cetuximab in BRAF V600E-mutated BTC, contributing to the limited but growing body of evidence supporting this approach. The response observed in our patient aligns with the biological rationale for simultaneously targeting the BRAF and MEK pathways, a strategy that has already proven effective in BRAF-mutated colorectal cancer. Given the poor prognosis typically associated with BRAF-mutated BTC, the initial tumor response in our case is particularly noteworthy. The duration of response and tolerability suggest that this triplet therapy may offer a viable treatment option for this molecular subset of BTC.

This case highlights the importance of comprehensive molecular profiling in BTC, as identifying actionable mutations can help guide more effective and individualized treatment decisions. Although initial disease control was achieved with encorafenib, binimetinib, and cetuximab, the tumor ultimately progressed. While re-biopsy and genomic reanalysis were not performed at the time of progression, several mechanisms of acquired resistance could be considered. These include the emergence of bypass signaling or alternative oncogenic drivers, such as KRAS mutations or activation of the PI3K pathway, as well as changes within the tumor microenvironment. These factors emphasize the complexity of resistance in BRAF V600E-mutated IHCC and highlight the need for further research, including longitudinal molecular monitoring, to better inform subsequent treatment strategies.

Several important considerations must be acknowledged. Experience with the encorafenib, binimetinib, and cetuximab combination in BTC remains limited, and larger studies are needed to confirm its efficacy and optimal dosage in this population. Additionally, the best timing for introducing targeted therapy within the treatment sequence, whether as first-line therapy or upon progression after standard chemotherapy, has yet to be defined. Understanding resistance mechanisms is also crucial for managing disease progression. The toxicity profile observed in our patient was manageable and aligned with existing data from colorectal cancer studies, emphasizing the importance of proactive monitoring and side effect management. However, the cost of this triplet regimen may present a financial barrier to access for some patients, which should be factored into treatment planning.

Large-scale genetic analyses have identified several recurrent mutations in BTC, including TP53, KRAS, SMAD4, CDKN2A/2B, and ARID1A [13-17]. In a study by Nakamura et al., actionable genetic alterations were found in about 40% of 260 Japanese patients with BTC [13], with site-specific mutations such as FGFR1, FGFR2, IDH1/2, BAP1, and ARID1A often seen in IHCC. Extrahepatic cholangiocarcinoma more commonly involves genetic abnormalities in PKA and HER2, while gallbladder cancer frequently harbors EGFR, HER2, and ERBB3 alterations [13]. These findings reinforce the need for molecular profiling to identify candidates for targeted therapy.

BRAF V600E mutations are detected in approximately 5-7% of BTCs and are especially prevalent in IHCC [5,6]. The phase II ROAR trial evaluated the efficacy of dabrafenib, a BRAF inhibitor, combined with trametinib, a MEK inhibitor, in patients with previously treated BRAF V600E-mutant BTC [7,18]. This basket trial enrolled 43 patients in the BTC cohort, 91% of whom had IHCC. The regimen demonstrated a response rate of 47% (central review), with a median progression-free survival of nine months and a median OS of 14 months. Based on these results, the National Comprehensive Cancer Network recommends dabrafenib plus trametinib for patients with BRAF V600E-mutant BTC [7].

At the time of treatment in our case, dabrafenib plus trametinib was not yet covered by insurance in Japan. After the discussion, the patient received encorafenib, binimetinib, and cetuximab. While clinical data on this triplet therapy in BTC remain limited to case reports, it has shown promising efficacy in BRAF V600E-mutated colorectal cancer. The toxicity profile in our patient was manageable and consistent with existing reports, suggesting this regimen may offer an alternative, particularly for patients intolerant to dabrafenib and trametinib due to adverse effects such as fever [7]. These findings support the need for further research into the BRAF/MEK/EGFR inhibition strategy for BTC.

Dabrafenib plus trametinib has demonstrated effectiveness in treating BRAF V600E-mutated solid tumors but is often associated with severe side effects, particularly fever [7]. Therefore, encorafenib, binimetinib, and cetuximab may be a viable alternative in patients unable to tolerate standard therapy. This case supports the rationale for larger, prospective studies to explore the role of this triplet therapy in BRAF-mutated BTC. Future research should focus on identifying predictive biomarkers, understanding mechanisms of resistance, and refining patient selection to optimize outcomes.

## Conclusions

This case illustrates the potential to extend targeted therapeutic strategies from well-studied malignancies to rare molecular subtypes. As the understanding of cancer biology deepens, treatment approaches may increasingly be guided by molecular features rather than traditional classifications based on tissue of origin. Although clinical reports remain limited, this case highlights the potential utility of encorafenib, binimetinib, and cetuximab in BRAF V600E-mutated BTC. Incorporating routine molecular profiling in the clinical management of BTC may help identify more patients who could benefit from targeted therapies.
